# Detection of chronic wasting disease prions in the farm soil of the Republic of Korea

**DOI:** 10.1128/msphere.00866-24

**Published:** 2025-01-30

**Authors:** Kyung-Je Park, Hoo-Chang Park, Yu-Ran Lee, Gordon Mitchell, Young Pyo Choi, Hyun-Joo Sohn

**Affiliations:** 1 WOAH Reference Laboratory for CWD, Foreign Animal Disease Division, Animal and Plant Quarantine Agency, Gimcheon, South Korea; 2 National and WOAH Reference Laboratory for Scrapie and CWD, Canadian Food Inspection Agency, Ottawa, Ontario, Canada; 3 Division of Research Strategy, Korea Brain Research Institute, Daegu, South Korea; University of Michigan, Ann Arbor, Michigan, USA

**Keywords:** prions, chronic wasting disease, soil, farm, detection, protein-misfolding cyclic amplification, Republic of Korea

## Abstract

Chronic wasting disease (CWD) is a highly contagious prion disease occurring in free-ranging and farmed cervids. CWD continues to spread uncontrolled across North America, and cases continue to be detected almost every year in the Republic of Korea. CWD-infected animals contaminate the soil by releasing infectious prions through their excreta, and shed prions accumulate and remain infectious in the soil for years. Given that the upper soil levels can become contaminated with prions and serve as infectivity reservoirs facilitating horizontal transmission of CWD, the ability to detect prions in the soil is needed for monitoring and managing CWD spread. Using the protein misfolding cyclic amplification (PMCA) technique, we investigated whether prions could be amplified and detected in farm soil experimentally exposed to CWD-infected brain homogenate as well as in the soil of CWD-affected farms. From each soil sample, we performed 10 serial extractions and used these 10 extracts as PMCA templates. Here, we show that prion seeding activity was detected in extracts from farm soil following 4 years of incubation with CWD-infected brain homogenate. More importantly, 13 of 38 soil samples collected from six CWD-affected farms displayed prion seeding activity, with at least one soil sample in each farm being PMCA positive. Mouse bioassays confirmed the presence of prion infectivity in the soil extracts in which PMCA seeding activity was detected. This is the first report describing the successful detection of prions in soil collected from CWD-affected farms, suggesting that PMCA conducted on serial soil extracts is a sensitive means for prion detection in CWD-contaminated soil.

**IMPORTANCE:**

Chronic wasting disease (CWD) is a highly contagious prion disease affecting free-ranging and farmed cervids. CWD continues to spread uncontrollably across North America, and multiple cases are detected annually in the Republic of Korea. Prions shed from CWD-infected animals remain infectious in the soil for years, serving as infectivity reservoirs that facilitate horizontal transmission of the disease. Therefore, the ability to detect CWD prions in soil is crucial for monitoring and managing the spread of the disease. In this study, we have demonstrated for the first time that prions in the soil of CWD-affected farms can be reliably detected using a combination of serial soil extraction and a prion amplification technique. Our data, in which at least one soil sample tested positive for CWD in each of the six CWD-affected farms analyzed, suggest that the approach employed in this study is a sensitive method for prion detection in CWD-contaminated soil.

## INTRODUCTION

Prion diseases are fatal neurological disorders impacting a range of mammalian species and include Creutzfeldt-Jakob disease in humans, bovine spongiform encephalopathy in cattle, and scrapie in sheep (1, 2). Prion diseases are characterized by the conversion of a monomeric cellular prion protein (PrP^C^) into an aggregated and pathological isoform (PrP^Sc^) (3, 4). PrP^Sc^ is thought to be the main component of the proteinaceous infectious agent or prion (5, 6) and can replicate in an auto-catalytic manner by binding to and converting PrP^C^ into the PrP^Sc^ conformer (7–9). While PrP^C^ is detergent-soluble and fully susceptible to protease digestion, PrP^Sc^ shows increased detergent insolubility and partial resistance to proteolytic degradation (10). The identification of amino-terminally truncated proteinase K (PK)-resistant core fragments of PrP^Sc^ following proteolytic treatment is a widely used diagnostic marker for prion infection (11, 12). Prions are notoriously difficult to fully inactivate through the routine decontamination procedures applied against most conventional pathogens (13–15), and they are extremely resistant to environmental degradation (16, 17).

Chronic wasting disease (CWD) is a contagious prion disease affecting multiple cervid species, including elk (*Cervus canadensis*), mule deer (*Odocoileus hemionus*), red deer (*Cervus elaphus*), sika deer (*Cervus nippon*), and moose (*Alces alces*) (18, 19). CWD can be transmitted horizontally via both direct and indirect routes, affecting not only farmed but also free-roaming animals (20, 21). Since the initial identification of CWD in 1967 in Colorado, USA (22), CWD has continued to expand in geographic range and prevalence. In North America, CWD has now been identified in more than 30 US states and 4 Canadian provinces (23). Additionally, multiple cases of CWD have also been reported in the Republic of Korea and Scandinavia (24, 25). In contrast to many other prion diseases where prions are largely confined to the central nervous system (CNS), CWD prions are found throughout the body, including lymphoid tissues, fat, urine, saliva, blood, and feces, as well as the CNS (26–29). CWD-infected animals contaminate the environment by releasing prions through their excreta, including saliva, urine, and feces (30, 31). Upon entering the environment, prions bind to soil and other environmental materials remaining infectious for years (32–36). The upper layer of soil becomes contaminated with prions and can serve as a long-term reservoir of CWD infectivity, facilitating horizontal transmission of the disease in the absence of direct contact between animals (37).

Since shed prions may be widely distributed by infected animals and remain infectious for extended periods of time, the ability to detect prions in the environment is essential for monitoring and managing CWD spread. Studies to reliably detect CWD in the environment have been performed primarily on soil but have also been applied to plants and other environmentally relevant surfaces (38–40). Commonly, *in vitro* amplification technologies, such as the protein misfolding cyclic amplification assay (PMCA), are employed due to their high sensitivity in prion detection (34, 41–43). One limitation of this approach is that CWD PrP^Sc^ bound to soil needs to be extracted for efficient replication in PMCA, given that PMCA reactions seeded with soil-bound CWD PrP^Sc^ were found to be significantly inhibited (44). While unbound PrP^Sc^ is thought to be the favored template for efficient replication in PMCA (44), it is known to be difficult to recover PrP^Sc^ from a variety of soil types, particularly when incubated for a long time (40, 45, 46).

In this study, we investigated whether CWD prions in the soil of Korean cervid farms can be detected through the combination of repeated extraction and PMCA analysis of these serial extracts. We show that prions can be detected in farm soil up to 4 years after being experimentally contaminated with a range of concentrations of CWD-affected brain homogenate. We also demonstrate that prion seeding activity is present in the soil of the Korean cervid farms in which CWD-positive animals have been identified. Our data suggest that the approach described in this study can be used to detect prions with high sensitivity in CWD-contaminated soil.

## MATERIALS AND METHODS

### CWD prion source

A male elk showing clinical symptoms consistent with CWD was culled at the age of 7 and diagnosed as CWD positive by the Animal and Plant Quarantine Agency (APQA) in the Republic of Korea. The whole brain of this CWD-positive elk was manually homogenized with mortar and pestle as a 10% (wt/vol) homogenate in 0.9% sterilized saline. The infectivity titer of this brain homogenate was calculated to be 10^5.6^ LD_50_ per gram of brain tissue, which was determined using TgElk mice described below. This CWD brain homogenate was used as a prion source for the experimental contamination of farm soil.

### Soil contamination with CWD prions

Experimental contamination of farm soil with CWD was performed as described previously with minor modifications (47). Briefly, soil was obtained from a CWD-free cervid farm run by a local veterinary authority in the Republic of Korea. The farm soil has a silt loam texture, which is known to be typical in Korean farmlands (48). For the contamination experiment, 28 grams of farm soil was moved to a plastic 50 mL conical screw cap tube (approximately 5 cm in depth) and irradiated as previously described to inactivate viruses and bacteria without having a substantial effect on prion infectivity. After being saturated with 5 mL of distilled water and left at room temperature for a further 16 hours, the soil in each tube was exposed to 3 mL of CWD-positive elk brain homogenate prepared at one of the following concentrations (wt/vol): 1%, 0.1%, 0.01%, or 0.001%. The addition of 3 mL of brain homogenate in each tube was repeated once a week for 4 months, while being kept at room temperature. Adding brain homogenate at a weekly interval was chosen to allow sufficient time for evaporation as occurs in natural conditions, and the 4-month period was based on the duration of clinical disease (49) and observations that prion shedding becomes more pronounced as the clinical phase approaches (26, 50, 51). Subsequently, the tubes containing CWD-contaminated soil were maintained at 22°C with a humidity of 50%–55% for up to 4 years. Approximately, 1 gram of soil in each tube was taken once a year for PMCA analysis. This 4-year incubation was selected based on reports that prion infectivity or seeding capacity of various strains can persist in the soil for several years (17, 40, 45, 52).

### Soil collection in cervid farms

Soil collection was conducted by personnel of the local veterinary authorities. Soil was collected from 25 cervid farms associated with CWD cases in 2016 in the Republic of Korea. While CWD-positive animals were detected in 6 farms, CWD was not identified in animals in the remaining 19 farms despite epidemiological involvement with the CWD-positive farms. CWD testing of these animals was conducted on obex and retropharyngeal lymph node tissues using the IDEXX HerdChek BSE-Scrapie Antigen Test. In each farm, soil was collected in four locations: the feeding trough, barn, pen, and path. In each location, soil was gathered into a zipper bag (27 × 30 cm) from more than five spots with at least 10 grams of soil taken per spot (5 grams per spot near the feeding trough). For the feeding trough, more than 5 grams of deposit per trough were also collected from the bottom of the container. The number of samples in each farm was determined based on the number of zipper bags that arrived at APQA for analysis.

### Soil extraction

To extract CWD prions from the soil, 0.5 gram of soil from each sample was moved into a 15 mL tube and then 3 mL of phosphate buffer solution (PBS) was added to the tube. Following 20-second vortex at maximal speed using Vortex-Genie 2 mixer with pop-off cup head (Scientific Industries, Inc.), the mixture was centrifuged at 1,000 × *g* for 5 minutes, and 500 µL of the supernatant was recovered and stored (first extract). Subsequently, 500 µL of new PBS was added to the 15 mL tube, and the mixture was vortexed in the same way. Following centrifugation at 1,000 × *g* for 5 minutes, 500 µL of the supernatant was recovered and stored (second extract). This step was repeated eight more times (3rd to 10th extracts). After the 10th extraction, the remaining 0.5 gram soil was resuspended in 1 mL PBS and used as a seed in PMCA analysis. The presence of CWD prion seeding activity was studied in the 10 extracts and the soil resuspension samples after serial extraction using the PMCA technique, as described below.

### Transgenic mice

A transgenic mouse line that expresses the elk prion protein (PrP) with methionine at position 132 was described previously (53). This transgenic mouse line homozygous for the elk PrP (hereafter referred to as TgElk) was kindly provided by Dr. R. Rubinstein (Institute for Basic Research in Developmental Disabilities, USA). The expression level of elk PrP in TgElk mice is estimated to be 2.5-fold higher than the level of PrP^C^ in background FVB/N mice (53).

### PMCA procedure

The brains of TgElk mice were used as a substrate for PMCA amplification of CWD prions. Brains were homogenized at a concentration of 5% (wt/vol) in PMCA conversion buffer (PBS containing 1% Triton X-100, 5 mM EDTA, 150 mM NaCl, 0.05% Digitonin, and cOmplete protease inhibitor cocktail [Roche]). Following clarification at 2,000 × *g* for 1 minute, the cleared brain homogenates were stored at −80°C until use. The 10 consecutive extracts and soil resuspension after serial extraction from each sample were used as seeds in PMCA reactions. As controls, 10% (wt/vol) brain homogenates obtained from CWD-affected elk and normal elk were used in this study. One hundred microliters of PMCA reaction mixture consisting of 80 µL substrate and 20 µL seed was loaded onto 0.2 mL thin-walled PCR tubes supplemented with two Teflon beads (2.38 mm diameter, McMaster, Los Angeles, CA, USA). The tubes were incubated in a water bath at 37°C for 16 hours and then placed in a microsonicator (Misonix model 4000) set at an amplitude of 75. For the first round of PMCA, the tubes were subjected to 56 cycles of 30-second sonication followed by 9-minute 30-second incubation in the water of the sonication bath at 37°C. Seventy microliters of the first-round PMCA products was taken to a new tube for future analysis. Subsequently, 70 µL of fresh substrate was added to the first-round tubes and submitted to the second round of PMCA cycles. This procedure was repeated one more time (third round PMCA). To assess the potential presence of PrP^Sc^, 5 µL of the PMCA products was digested with 200 µg/mL PK for 1 hour at 37°C and analyzed by Western blotting.

### Bioassay

TgElk mice were used to assess the potential presence of prion infectivity in the extracts and soil resuspensions. Regarding the 10 serial extracts per sample, only extracts positive for PrP^Sc^ after the third round of PMCA were merged into one inoculum and examined by mouse bioassay. When all 10 extracts were negative for PrP^Sc^ after the third round of PMCA, they were combined into one inoculum and investigated by mouse bioassay. Soil resuspension samples (0.5 gram of soil resuspended in 1 mL PBS) after 10 serial extraction steps were analyzed without further dilution. Following anesthesia with isoflurane in oxygen, 8-week-old female TgElk mice were intracranially injected with 20 µL of inoculum. As controls, 10% brain homogenates obtained from CWD-affected red deer and normal red deer were included in this study. Six mice were used per inoculum. The inoculated mice were monitored daily for the onset of clinical signs. Clinical signs observed in these mice included rough coat, sticky eyes, hunched back, limb paresis, convulsion, depression, and emaciation. When they showed more than three of these symptoms over 1 week, the mice were euthanized and necropsied. One half of each brain was fixed in 10% neutral buffered formalin for histopathology, and the other half was homogenized in PBS at 10% (wt/vol) for western blot analysis after 30-minute PK treatment at 50 µg/mL.

### Western blot analysis

The PK-digested PMCA products or mouse brain homogenates were mixed with 4× NuPAGE LDS sample buffer (Invitrogen) to a final concentration of 1×. Following 10-minute incubation in a heating block set at 100°C, proteins were separated in 12% Bis-Tris gels (Invitrogen) and then blotted onto polyvinylidene difluoride membranes (Millipore). The membranes were blocked with 0.2% I-Block (Applied Biosystems) in TBST (20 mM Tris-HCl [pH 7.4], 150 mM NaCl, and 0.1% Tween 20). Subsequently, the membranes were incubated with rabbit anti-PrP serum raised against a synthetic bovine PrP 106-122 peptide (CTHGQWNKP SKPKTNMK) and then with horseradish peroxidase-labeled anti-rabbit IgG antibody (KPL 074-1516) at a dilution of 1:5,000. The blots were developed with Supersignal West Pico PLUS Chemiluminescent Substrate. The molecular weights of PK-resistant core fragments of PrP^Sc^ were determined by reference to Amersham ECL Rainbow molecular weight markers (RPN800E, GE Healthcare).

## RESULTS AND DISCUSSION

To investigate whether CWD prions in the soil of Korean cervid farms can be detected using PMCA, we first tested farm soil that had been experimentally contaminated with varying concentrations of CWD-positive brain homogenate (1%, 0.1%, 0.01%, or 0.001%). Among these four concentrations, PrP^Sc^ was expected to be detectable using PMCA on farm soil exposed to the highest concentration of brain homogenate (1%) based on our previous study (47). The lowest homogenate concentration (0.001%) was selected to be comparable to the level of prion infectivity excreted by a CWD-infected deer during a single urination event, given the reported CWD prion levels in urine (42, 50) and their daily urine output and urination frequency (54). In this case, 3 mL of the 0.001% brain homogenate was delivered weekly to the farm soil and was estimated to contain 12 LD_50_ of prion infectivity. The farm soils contaminated with CWD brain homogenates were incubated at 22°C for up to 4 years while being sampled once a year.

**Fig 1:**
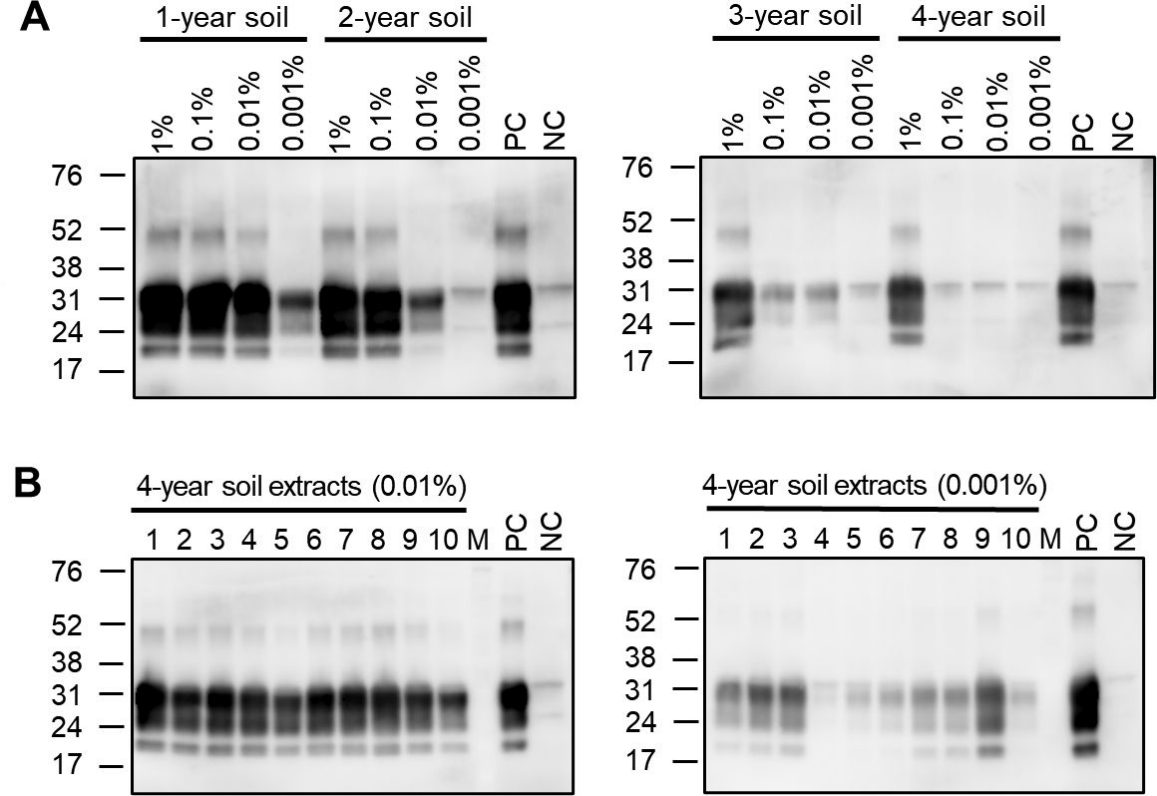
Detection of prions in soil experimentally exposed to CWD-positive brain homogenates. Soil collected from a CWD-free elk farm was exposed to various concentrations of CWD brain homogenate (range: 1%–0.001%) and then incubated at 22°C for up to 4 years while being sampled once a year for analysis. Each soil sample was subjected to 10 repeated PrP^Sc^ extractions as described in Materials and Methods. The presence of PrP^Sc^ in each soil sample following 10 serial extractions (**A**) as well as in the 10 extracts obtained from soil exposed to either 0.01% or 0.001% CWD brain homogenates for 4 years (**B**) was assessed following the third PMCA round. The third round PMCA products were treated with PK and analyzed by Western blotting using anti-PrP rabbit serum raised against bovine PrP 106-122 peptide. Molecular weight markers are shown on the left.

Given that unbound PrP^Sc^ is preferred over soil-bound PrP^Sc^ as the PMCA template (44), we performed 10 repeated PrP^Sc^ extractions from the CWD-contaminated soil and then investigated the presence of PrP^Sc^ using PMCA in the 10 extracts as well as in the soil remaining after these repeated extractions. PMCA results were interpreted after three rounds (47). We first tested whether PrP^Sc^ could be identified in the soil that had experienced 10 serial extractions. In the soil incubated for 1 year, PrP^Sc^ was identified following PMCA amplification irrespective of the concentrations of CWD brain homogenate used for soil contamination (Fig. 1A). In comparison, after 4 years of incubation, PrP^Sc^ was only readily detectable in the soil exposed to the 1% CWD brain homogenate (the highest concentration applied in this study) (Fig. 1A). While PrP^Sc^ in the soil exposed to 0.01% CWD brain homogenate was identifiable after up to 3 years of incubation, PrP^Sc^ in the soil exposed to 0.001% homogenate was detectable only after 1 year of incubation (Fig. 1A). The farm soil used for experimental contamination with CWD prions did not show any PMCA seeding activity, indicating that the soil obtained from this farm was not previously contaminated with CWD prions (Fig. S1). Our results suggest that the PrP^Sc^ remaining bound to soil following serial extraction became less and less efficient in supporting PMCA reactions, with incubation times increasing from 1 to 4 years. Therefore, soil samples contaminated with low levels of CWD prions may not be optimal for use as seeds in PMCA analysis if contamination occurred several years earlier.

Next, considering that unbound PrP^Sc^ is regarded as the preferred template for efficient PMCA replication (44), we aimed to investigate whether PrP^Sc^ could be amplified and detected in serial extracts from the CWD-contaminated soil in which PrP^Sc^ was undetectable by soil-based PMCA. To explore this possibility, we examined two sets of 10 serial extracts obtained from the soil samples incubated for 4 years following exposure to either 0.01% or 0.001% CWD brain homogenates. As shown in Fig. 1B, PrP^Sc^ was detected in both sets of extracts, despite the absence of detectable PrP^Sc^ in extracts from the soil sample exposed to the 0.001% homogenate. Our results suggest that PrP^Sc^ could be desorbed from the 4-year soil at a level high enough to support PMCA amplification even when contaminated with a 0.001% CWD brain homogenate (the lowest concentration applied in this study). Thus, the application of serial extracts as PMCA seeds enabled us to amplify and detect PrP^Sc^ in farm soil that had been exposed to very low levels of CWD brain homogenate and incubated for 4 years, which was not achievable when soil samples were used as PMCA seeds.

**Fig 2:**
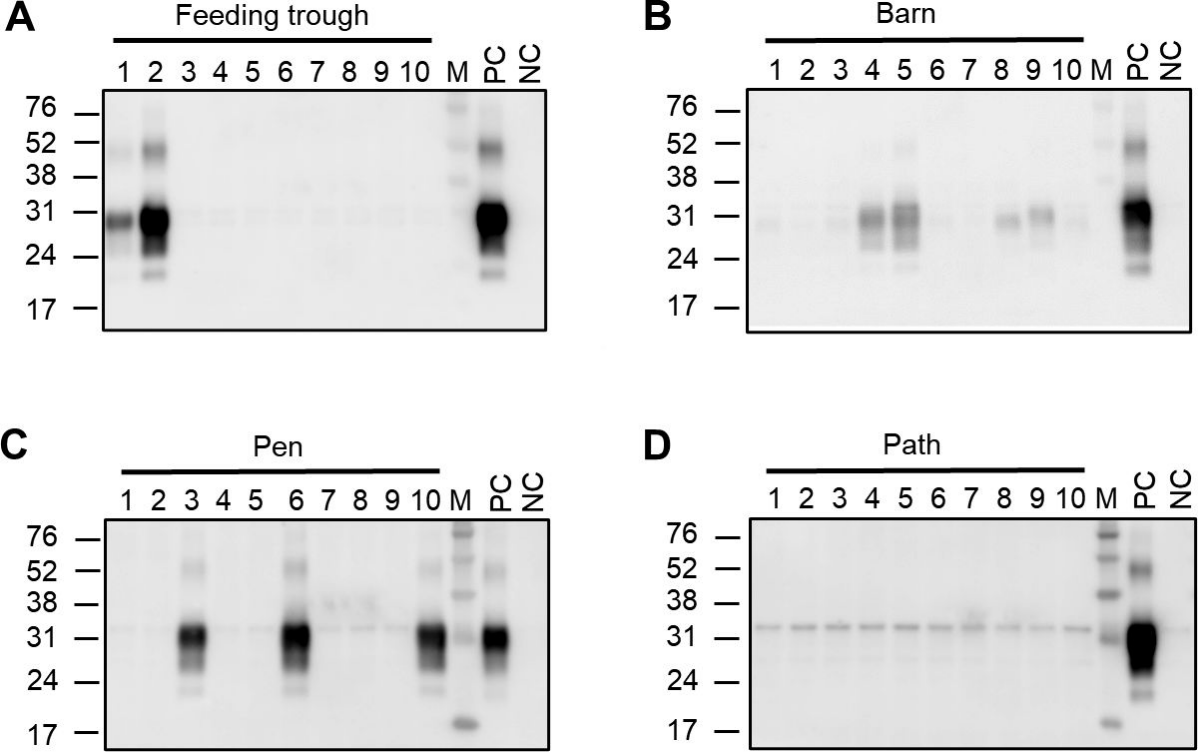
Detection of prions in the soil of a farm affected with CWD. Soil samples were collected from the feeding trough (**A**), barn (**B**), pen (**C**), and path (**D**) in a cervid farm where animals were found to be positive for CWD. Each soil sample was subjected to 10 repeated PrP^Sc^ extractions as described in Materials and Methods. The presence of PrP^Sc^ in the 10 extracts from each soil sample was assessed following three PMCA rounds. The third round PMCA products were treated with PK and analyzed by Western blotting using anti-PrP rabbit serum raised against bovine PrP 106-122 peptide. Molecular weight markers are shown on the left.

With the improved sensitivity observed using serial soil extracts, we then proceeded to use this method to examine soil samples obtained from 25 cervid farms associated with CWD cases in 2016 in the Republic of Korea. CWD-positive animals were detected in 6 of 25 farms. We did not identify any CWD-positive animals in the remaining 19 cervid farms despite their epidemiological links to the CWD-positive farms. In each farm, soil was collected from up to four different locations: the feeding trough, barn, pen, and path. We obtained 10 extracts from each of the farm soil samples and then analyzed all extracts by PMCA. Each soil sample was classified as CWD positive if a PrP^Sc^ signal was identified in any of the 10 extracts following three PMCA rounds. In the analysis of a total of 38 soil samples from six CWD-positive farms, at least one soil sample from each of the CWD-positive farms provided positive PrP^Sc^ signals in PMCA. While 50% of soil samples from the feeding trough or the barn exhibited PMCA seeding activity, none of the path samples were positive for PrP^Sc^ after the third PMCA round (Table 1). In the case of samples from the pen, two out of seven soil samples displayed positive PrP^Sc^ signals. Collectively, a sample positivity rate of 34.2% was observed when all 38 soil samples from four different locations on CWD-positive farms were combined (Table 1). Notably, in contrast to the results from soil experimentally exposed to CWD, PrP^Sc^ was less consistently identified in the soil sample extracts from CWD-positive farms. While PrP^Sc^ recovered from the soil around feeding troughs appeared early in a few extracts (Fig. 2A), PrP^Sc^ recovered from the soil around barns or pens was detected in intermediate and late extracts in a less predictable pattern (Fig. 2B and C).

**TABLE 1:** PMCA screening results in soil samples collected from 25 cervid farms

Classification^*a*^	Farm^*b*^	Soil source				
		Feeding trough^*c*^	Barn^*c*^	Pen^*c*^	Path^*c*^	Total^*c*^
CWD positive	6/6 (100%)	5/10 (50%)	6/12 (50%)	2/7 (28.6%)	0/9 (0%)	13/38 (34.2%)
CWD negative	2/19 (10.5%)	1/27 (3.7%)	1/28 (3.6%)	1/26 (3.8%)	0/16 (0%)	3/97**^*d*^** (3.1%)

^*a*^ Classification of each farm into CWD-positive or CWD-negative status was determined based on CWD testing of farm animals. While CWD-positive animals were detected in 6 of 25 farms, animals available for testing in the other 19 farms were all negative for CWD. There was no animal to be tested on one farm as it was not operational at the time of the investigation. We classified this farm as being CWD negative in the table.

^*b*^ Number of farms where one or more soil samples were PMCA positive/number of farms where soil samples were collected for PMCA analysis.

^*c*^ Number of soil samples positive in PMCA/number of total soil samples examined by PMCA.

^*d*^ Two of the three PMCA-positive samples (one barn soil sample and one pen soil sample) were collected from the farm that was not operational at the time of the investigation.

**Fig 3:**
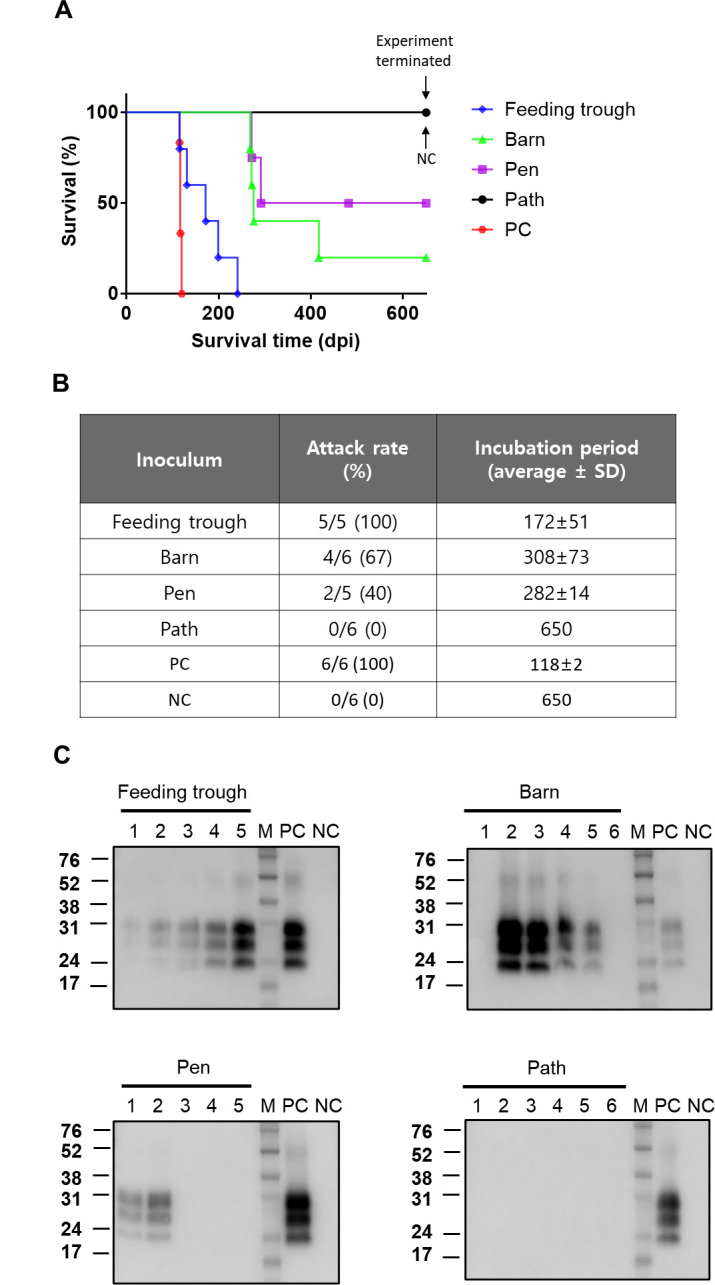
Animal bioassays to evaluate the presence of CWD infectivity in the soil of a farm affected with CWD. Soil samples were collected from the feeding trough, barn, pen, and path in a CWD-positive cervid farm. Each soil sample was subjected to 10 serial PrP^Sc^ extractions. The resultant extracts from each soil sample were merged into one inoculum as described in Materials and Methods, and the inoculum was intracerebrally inoculated into TgElk mice. A 10% brain homogenate prepared from CWD-infected or normal deer was included as positive control (PC) and negative control (NC), respectively. Mice that died within a week after inoculation were excluded from data analysis. Animal experiments were terminated at 650 days post-inoculation (dpi), and the remaining animals were sacrificed. Survival curves (**A**) and attack rates and incubation periods (**B**) for the TgElk mice injected with soil extracts of four different locations. Attack rates are expressed as the number of CWD-positive animals divided by the total number of animals injected with inoculum. (**C**) Following treatment with PK, the presence of PrP^Sc^ in mouse brains was assessed by Western blotting using anti-PrP rabbit serum raised against bovine PrP 106-122 peptide. Molecular weight markers are shown on the left.

Next, we examined whether the soil extracts were carrying prion infectivity. This possibility was explored utilizing soil extracts described in Fig. 2, where four soil samples came from the same CWD-positive farm. Regarding extracts from the trough, barn, and pen samples, the individual extracts displaying positive signals in PMCA were combined into one inoculum. In the case of the path extracts, all 10 extracts were combined into one inoculum. The TgElk mice that were intracerebrally challenged with these combined extracts manifested disease with varying degrees of attack rate and incubation period. A complete attack rate and the shortest incubation period were seen for mice treated with the trough soil inoculum (Fig. 3A and B). In comparison, inocula prepared from extracts of barn or pen soil samples induced disease but with much longer incubation periods and incomplete attack rates (Fig. 3A and B). In Western blot analysis, PrP^Sc^ was detected only in mice euthanized due to clinical signs consistent with prion infection (Fig. 3C). TgElk mice challenged with the PMCA-negative path inoculum or normal deer brain homogenate remained healthy until sacrificed at the experimental endpoint (650 days post-inoculation) and did not display any detectable PrP^Sc^ in their brains. Our bioassay results demonstrate that CWD prion infectivity in the soil was recovered into the extracts, which is consistent with the PMCA results. Altogether, our results confirm that PrP^Sc^ bound to soil in CWD-contaminated farms can be extracted and amplified to detectable levels using PMCA.

We then analyzed the soil samples obtained from 19 CWD-negative farms whose disease status was determined following regulatory testing of these herds for CWD. Among the total of 97 soil samples, 3 samples from two farms were found to be positive for CWD by PMCA (Table 1). An epidemiological investigation determined these two farms had received animals from one of the CWD-positive farms. Importantly, no animals were remaining to be tested for CWD in one of the two farms as it had ceased operation at the time of investigation. In the other farm, all 10 animals tested were negative for CWD. However, further investigation into this second farm revealed that four animals had been found dead in the last several months while showing clinical signs suspicious for CWD, but they had not been submitted for CWD testing. Our results from these particular “CWD-negative” farms further suggest that the approach employed in this study may be highly efficient in detecting prions in the soil.

CWD prions released in excreta, birthing fluids, placenta, or carcasses can accumulate in the environment while retaining infectivity (26, 27, 37, 55). Due to their extreme resistance to environmental degradation, prions deposited in the soil and other environmental materials can serve as long-lasting reservoirs for the horizontal transmission of CWD (35, 37). Accordingly, to control and manage CWD spread, it is important to be able to detect CWD prions in environmental components. Efforts to detect CWD prions in various environmental sample types, such as environmental surfaces, water, and parasites, have been successful using sensitive amplification techniques (36, 38, 39, 43, 56–58). However, applying similar approaches to soil has been less successful, often associated with the inefficient recovery of soil-bound PrP^Sc^ (40, 44). Refinements to extraction techniques have improved soil detection capabilities, as demonstrated here and recently by Kuznetsova et al. (59), who reported on the detection of PrP^Sc^ in soil from regions with a high prevalence of CWD. In this study, for the first time, we have shown that PrP^Sc^ in the soil of CWD-affected farms can be amplified and detected using PMCA.

The farm soil used for experimental CWD contamination has a silt loam texture (60% silt, 24.3% clay, and 15.7% sand) (47). Given that this type of soil is known to be representative of Korean farmlands (48), cervid farm soil samples examined in this study are considered to have similar soil properties to the experimentally CWD-contaminated sample. While the extraction of PrP^Sc^ from soil is needed for efficient replication in PMCA due to the preference for unbound PrP^Sc^ as a template, it is also known that the recovery of PrP^Sc^ could be relatively inefficient in clay-rich soil (46, 60). Thus, it remains to be investigated if our approach could be successfully applied for the detection of CWD prions in different types of soil, including clay-rich soils. We also observed the gradual decline and eventual disappearance of the PMCA signal as the incubation time of experimentally contaminated soil increased from 1 to 4 years. This observation may reflect the greatly reduced efficiency of PMCA amplification of soil-bound prions after lengthy incubation (52). Alternatively, this result might be attributed to the degradation of CWD prions in the soil by organic matter compounds, such as humic acids, as previously described (61, 62). Moreover, in contrast to the stable conditions in the laboratory where CWD-contaminated soil was incubated in this study, constant physical changes, such as temperature and moisture fluctuations, in the environment may further affect the stability of CWD prions in soil. These factors are believed to have collectively contributed to the challenges in detecting CWD prions in natural soil using PMCA. Altogether, our data suggest that PMCA conducted on serial soil extracts can overcome some of these challenges and serve as an effective tool to sensitively detect prions in soil.

## SUPPLEMENTAL MATERIAL

Figure S1 msphere.00866-24-s0001.tiff Western blot analysis of soil from a CWD-free cervid farm.

Legend msphere.00866-24-s0002.docx Figure S1 legend.

ASM does not own the copyrights to Supplemental Material that may be linked to, or accessed through, an article. The authors have granted ASM a non-exclusive, world-wide license to publish the Supplemental Material files. Please contact the corresponding author directly for reuse.
