# Supplementary figures and images for: Detection of chronic wasting disease prions in the farm soil of the Republic of Korea
Source: mSphere. 2025 Jan 30;10(2):e00866-24. doi: 10.1128/msphere.00866-24 (PMC11852723; doi:10.1128/msphere.00866-24)

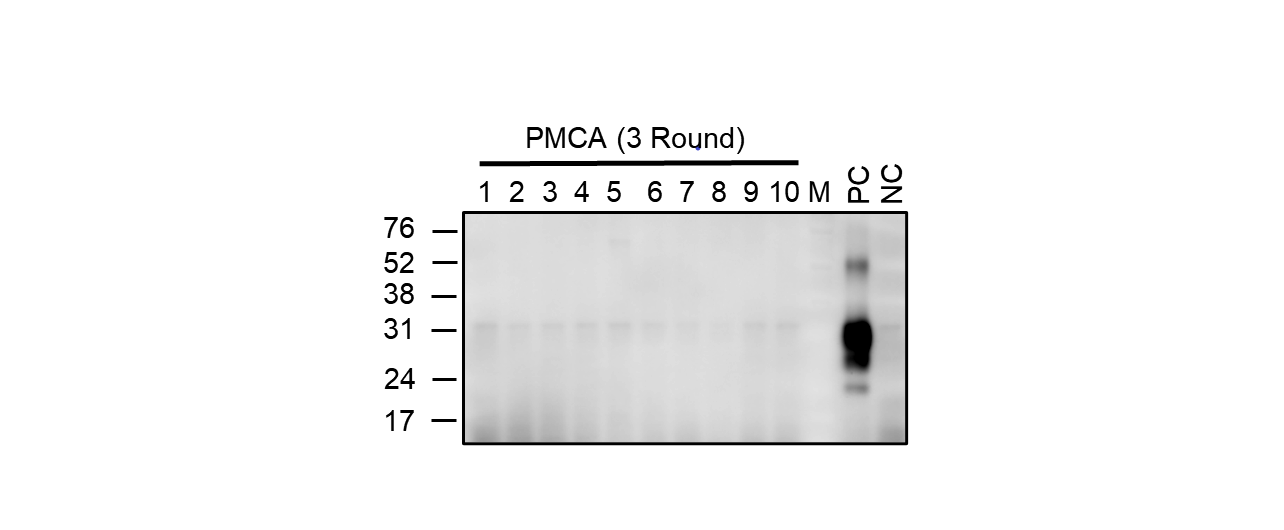

Supplement: Figure S1 — Western blot analysis of soil from a CWD-free cervid farm. [file msphere.00866-24-s0001.tiff]
