# Supplementary material for: Detection of chronic wasting disease prions in the farm soil of the Republic of Korea
Source: mSphere. 2025 Jan 30;10(2):e00866-24. doi: 10.1128/msphere.00866-24 (PMC11852723; doi:10.1128/msphere.00866-24)
Supplement: Legend — Figure S1 legend. [file msphere.00866-24-s0002.docx]

**Supplementary Figure 1. Western blot analysis of soil from a CWD-free cervid farm.** Soil was collected from a CWD-free cervid farm run by a local veterinary authority in the republic of Korea. Following the transfer of 28 grams of soil to a plastic 50 mL conical screw cap tube, soil was saturated with 5 mL of distilled water and left at room temperature for 16 hours. Soil was then subjected to 10 repeated PrP^Sc^ extractions as described in the method section. The presence of PrP^Sc^ in the 10 extracts was assessed following the third PMCA round. The third round PMCA products were treated with PK and analysed by Western blotting using anti-PrP rabbit serum raised against bovine PrP 106-122 peptide. Molecular weight markers are shown on the left.
